# Similar Spectral Power Densities Within the Schumann Resonance and a Large Population of Quantitative Electroencephalographic Profiles: Supportive Evidence for Koenig and Pobachenko

**DOI:** 10.1371/journal.pone.0146595

**Published:** 2016-01-19

**Authors:** Kevin S. Saroka, David E. Vares, Michael A. Persinger

**Affiliations:** 1 Behavioural Neuroscience Laboratory, Laurentian University, Sudbury, Ontario, Canada P3E 2C6; 2 Human Studies Program, Laurentian University, Sudbury, Ontario, Canada P3E 2C6; 3 Biomolecular Sciences Programs, Laurentian University, Sudbury, Ontario, Canada P3E 2C6; University of British Columbia, CANADA

## Abstract

In 1954 and 1960 Koenig and his colleagues described the remarkable similarities of spectral power density profiles and patterns between the earth-ionosphere resonance and human brain activity which also share magnitudes for both electric field (mV/m) and magnetic field (pT) components. In 2006 Pobachenko and colleagues reported real time coherence between variations in the Schumann and brain activity spectra within the 6–16 Hz band for a small sample. We examined the ratios of the average potential differences (~3 μV) obtained by whole brain quantitative electroencephalography (QEEG) between rostral-caudal and left-right (hemispheric) comparisons of 238 measurements from 184 individuals over a 3.5 year period. Spectral densities for the rostral-caudal axis revealed a powerful peak at 10.25 Hz while the left-right peak was 1.95 Hz with beat-differences of ~7.5 to 8 Hz. When global cerebral measures were employed, the first (7–8 Hz), second (13–14 Hz) and third (19–20 Hz) harmonics of the Schumann resonances were discernable in averaged QEEG profiles in some but not all participants. The intensity of the endogenous Schumann resonance was related to the ‘best-of-fitness’ of the traditional 4-class microstate model. Additional measurements demonstrated real-time coherence for durations approximating microstates in spectral power density variations between Schumann frequencies measured in Sudbury, Canada and Cumiana, Italy with the QEEGs of local subjects. Our results confirm the measurements reported by earlier researchers that demonstrated unexpected similarities in the spectral patterns and strengths of electromagnetic fields generated by the human brain and the earth-ionospheric cavity.

## Introduction

In 1954 Von W. O. Schumann and H. Koenig [1] reported reliable and predictable peaks of frequencies that were consistent with the model of an earth-ionospheric resonance. The possibility that the electrical components of the time-varying electrical potentials produced by the brain may occasionally overlap and become synchronous with ultra-low frequency (ULF) electromagnetic activity occurring within this resonant cavity was originally observed and reiterated by Koenig and his colleagues [2,3]. In their publication they noted qualitative congruencies between the waveforms of electroencephalographic activity recorded from the scalps of human subjects and patterns of naturally occurring electromagnetic activity (Type I and II signals) that are generated by global lightning, particularly in tropical regions close to the equator where lightning is observed year round. This observation is now supported by additional quantifications showing that the space-time parameters of signals measured from both the earth-ionosphere and brain electrical activity are complimentary. In particular the Schumann resonances, which are traditionally defined by spectral peaks at approximately 8, 14, 20, 26, and 33 Hz [4], show remarkable consistency with electroencephalographic activity in terms of frequency and intensity; both exhibit average magnetic field intensities of about 1–2 picoTeslas and when the average cortical thickness of about 3 mm is accommodated both exhibit electric field intensities approaching .1 to 1 mV/m.

The apparent relationship between the Schumann resonance and brain activity has been assessed theoretically. Nunez modeled the skull-brain cavity after the earth-ionospheric cavity and predicted mathematically that the dominant resonant frequency of the brain would be about 10 Hz [5]; this peak frequency decreased as a function of increasing skull size of the individual [6]. Persinger, applying the concepts of scale-invariance, showed that the current densities of action potentials propagating along an axon were similar to those of lightning strikes suggesting that a fractal relationship between processes occurring within the brain were reflective of processes occurring over the entire planet [7].

Many research groups have quantitatively assessed coupling of activities occurring globally as geomagnetic perturbations within local regions electrically within the human brain. Replicating observations made by Babyayev and Allahverdiyeva [8], Mulligan and colleagues [9] described that theta (4–7 Hz) activity within the right prefrontal sensor was positively correlated with terrestrial atmospheric power, an indicator of the strength of the steady-state magnetic field of the earth. Later, Saroka et al. [10] showed that both bi-temporal coherence and parahippocampal activity was positively correlated with the strength of geomagnetic displacement, the *k-index*. More detailed analyses indicated that the strength of the relationship between posterior left-right temporal lobe coherence and geomagnetic activity was strongest for coherences at 7.81 and approximately 20Hz, or stated alternatively the first and third harmonic of the Schumann resonance.

To test direct synchrony between magnetic processes occurring in the earth-ionosphere cavity and the human brain, Saroka and Persinger [11] measured simultaneously the Schumann resonance and brain electrical activity of a single individual that was sitting quietly outside with eyes-closed. Results of the analysis indicated the presence of transient periods of ‘harmonic synchrony’ that appeared when cross-channel coherence was computed between the caudal root-mean-square signal derived from the brain and the extremely-low frequency (ELF) magnetic activity occurring in the proximal environment. These periods of harmonic synchrony lasted approximately 200–300 msec and consisted of simultaneous coherence within the 7–8, 13–14 and 19–20 Hz bands. The coherence magnitudes were similar in to those reported earlier by Pobachenko and colleagues [12].

Our approach is that the human cerebrum is a functional, dynamic dipole whose voltage, or potential difference, is reflected as more or less steady-state potentials in the order of 10 to 30 mV [13] and extremely low frequency time-varying components whose average intensities are about three orders of magnitudes less (μV range). Because the human cerebral volume is not a sphere but more typical of an elliptoid there should be a slight difference in average μV intensity that reflects this ratio. The relatively fixed volume and surface area of the cerebrum and rostral-caudal bulk velocity of ~4.5 m·s^-1^ should generate constant standing waves [6,14] with parameters much like the fundamental Schumann resonance of ~7.5 to 8 Hz [3,15,16,17] which is the resonance solution for the velocity of light (3·10^8^ m·s^-1^) and the earth’s fixed circumference (4·10^7^ m). The Schumann resonances were likely present during the origin of living systems [18].

The purpose of the present study was to 1) to explore the potential manifestation of the Schumann resonances within the electro-cortical activity of a large sample of individuals by quantitative electroencephalographic assessments using various methodologies and 2) to replicate the findings observed earlier showing an enhanced synchrony between human cortical activity and the Schumann resonance when the later was measured locally in Sudbury, Canada as well as distally in Cumiana, Italy.

## Materials and Methods

### Study 1-Intrinsic Schumann Resonance in Human Brain Activity

#### Subjects

The subjects in this study were 184 individuals, measured singly, who had participated in various experiments within the laboratory between 2009 and 2013. Some subjects were measured more than once so that the total numbers of records were 237. As part of the laboratory protocol, eyes-closed measurements were collected at the beginning of each experiment before testing. While exact indices of age of the participants were not available, the majority of the individuals included in this study were university students between 19 and 25 years of age. Some of the individuals had been referred to the third author’s private practice for psychometric and electroencephalographic assessment (N = 45). The proportion of men (N = 109) and women (N = 128) were similar. Most measurements were completed within a commercial acoustic (echoic) chamber under dim light conditions, otherwise measurements were obtained in quiet rooms on the University campus from participants who volunteered for participation in 4^th^-year thesis projects.

#### Equipment and Recordings

Brain electrical activity was monitored using a Mitsar 201 amplifier equipped with a 19-channel Electro-Cap International. Measurements from 19 sites (Fp1, Fp2, F7, F3, Fz, F4, F8, T3, C3, Cz, C4, T4, T5, P3, Pz, P4, T6, O1, O2) consistent with the International Standard of Electrode Placement were obtained. Impedance for all measurements was maintained below 5 kOhms. The data recorded from the amplifier was delivered to a Dell laptop equipped with WinEEG v.2.8 which produced a digital copy of the recorded voltages. Sixteen-second epochs of eyes-closed data were extracted from each participant and exported into MATLAB software for further filtering and processing.

While most data collected with the amplifier were obtained using a sampling rate of 250 Hz, some measurements were collected with a sampling rate of 500 Hz. To insure homogeneity across subjects, data that were collected using a sampling rate of 500 Hz was re-sampled to 250 Hz using the *resample*.*m* function within the MATLAB platform. The data for each subject was then filtered between 1.5 and 40 Hz using the *eegfiltfft*.*m* function within the freely available EEGLab toolbox [19]. The function uses an inverse FFT algorithm to band-pass filter raw measurements within a specified frequency range. We have found qualitatively and quantitatively that this filtering algorithm produced identical results independent of whether the segment length was 16 seconds or 120 seconds in duration; the correlation between the raw voltage recordings was 0.996. Once filtered, the data were submitted to spectral analysis, using the *spectopo*.*m* function, which computed spectral density within discrete frequencies for the Fp1 O2 T3 and T4 sensors using Welch’s periodogram method employing a window size of 2048 (8.2 seconds) to maximize spectral resolution and a Hamming window with 50% overlap between windows.

These data was then imported into SPSS for further analysis and for the computation of mean absolute potential difference along the rostral-caudal (Fp1-O2) axis as well as between the left-right temporal (T3 and T4) sensors. Absolute differences between rostral-caudal and left and right temporal sensors were also obtained by subtracting the absolute raw voltages (independent from spectral density) from the extracted EEG record for each 4-millisecond point interval over 16 seconds.

#### Random Sample of 10 Brain Measurements

To appreciate the difference between a sub-sample of the total population and the total population, 16 s of QEEG data (Fp1 02, T3 T4) for 10 subjects were randomly selected using a random number generator. Z-scores for the μV^2^∙Hz^-1^ values for the rostral-caudal, left-right measures were obtained first in order to minimize individual differences in absolute baseline values. Spectral analyses from SPSS 16 (a different algorithm from the one applied in the previous section) were then applied to each of the two measures for each of the 10 subjects. The frontal spectral density scores were then subtracted from the occipital spectral density scores for each subject. The z-scores for each of these new means were calculated so that there would be a standardized score for these differences to compare directly across individuals. The average values for the two orthogonal measures were calculated.

In order to examine the degree of individual differences of the spectral analyses each subject’s results were assessed visually for the peak spectral density within the 10 Hz range. Because the Δf (increment of frequency) within this frequency range for 250 Hz samples is fractional (less than an integer, i.e., about .01 Hz), the band width of the peak spectral density could be inferred. This was discerned by direct inspection of the quantitative values where the decline in z-scores on either side of the peak was conspicuous and greater than 2 standard deviations for the interval.

#### Discerning the Schumann Resonance Signature in the Brain

After re-sampling and re-filtering between 1.5 and 40 Hz for the 184 individuals by eegfiltfft.m, the anterior, middle, and caudal root mean square signals were derived from integrating frontal (Fp1,Fp2,F7,F3,Fz,F4,F8), middle (T3,C3,Cz,C4,T4) and caudal (T5,P3,Pz,P4,T6,O1,O2) sensors. These derived signals were spectral analyzed by spectropo.m with a window size equal to 2048 FFT points to maximize spectral resolution employing the same spectral analysis parameters mentioned above. Other researchers, e.g., Abeyuriya et al [20], have recently employed the same approach to discern non-linear harmonics of sleep spindles in human electroencephalographic recordings.

Because of a connection between activity within the parahippocampus and naturally-occurring geomagnetic activity established in a previous publication [10] and its prominent role as an integrator of experience before long-term memory processes within the hippocampus, inferences of left and right parahippocampal current source density (μA·mm^-2^) were computed in sLORETA [21] within the classical frequency bands used in more conventional electroencephalographic studies [i.e. delta (1.5–4 Hz); theta (4–7 Hz); low-alpha (7–10 Hz); high-alpha (10–13 Hz); beta-1 (13–20 Hz); beta-2 (20–25 Hz); beta-3 (25–30 Hz); gamma (>30 Hz)]. Corradini and Persinger [22] have found that source localization performed with 19 sensors within a clinical population was able to localize the source of infarctions and closed-head injuries and was validated against accepted psychometric inferences of the functioning of these regions. We employed the exact same methodology for computing the current source density for the parahippocampal regions as in a previously published paper [10]. Each of the 237 16-second recordings were entered into sLORETA. First, the 19-channel series was translated into the frequency-domain using the *EEG to Cross-Spectrum* function and computed cross-spectral densities in the defined frequency bands [i.e. delta (1.5–4 Hz); theta (4–7 Hz); low-alpha (7–10 Hz); high-alpha (10–13 Hz); beta-1 (13–20 Hz); beta-2 (20–25 Hz); beta-3 (25–30 Hz); gamma (>30 Hz)]. Next the *Cross-Spectrum to sLORETA* function was used to compute current source density; the transformation matrix used here was defined by the 19-channel array produced within the software. Finally inferences of left (MNI-co-ordinates: X = -28, Y = -40, Z = -12) and right (X = 28, Y = -40, Z = -12) parahippocampal current source density for each of the frequency bands (for each participant) were extracted by using the *sLORETA to ROI* function using a customized ROI seed file.

The same epochs of electroencephalographic activity used in the sLORETA analyses were also imported into MapWin software [23] for the computation of microstates and their related parameters (i.e. duration of microstate, occurrence of microstate class, etc.). The strategies involved in producing microstates involves clustering of topographic maps based upon voltage. Because of the ‘stochastic’ nature of EEG signals, only those maps that exceed a certain threshold of ‘signal to noise’ are included [24]. The criterion for this threshold is defined by global field potential (GFP); only those maps with elevated GFP peaks that are high are considered for clustering. This equation is expressed as a spatial standard deviation and can be modeled mathemtaically as [24]:

$$GFP=(\sum {\left(v(t)-V{\left(t\right)}^{2}/n\right)}^{1/2}$$ (1)

where v is the voltage at a given channel, t is the time point of interest, and V is the mean voltage across all channels. These maps are then entered into a k-means clustering algorithm within the MapWin software itself and classes of microstate maps are produced irrespective of polarity. Effectively the resultant ‘classes’, or clusters, are the mean centers for each channel interpolated onto a 5x5 matrix with approximate locations of channels identified. The montage in this case was:

$$\begin{array}{ccccc} & Fp1 & & Fp2 & \\ F7 & F3 & Fz & F4 & F8\\ T3 & C3 & Cz & C4 & T4\\ T5 & P3 & Pz & P4 & T6\\ & O1 & & O2 & \end{array}$$

The 16-second epochs for each of the 237 available records were segmented into 4x4 second segments for later averaging. Then, microstate computations were computed separately for each participant utilizing the following methods. First, each of the 4 segments were imported into MapWin where a digital filter between 2–20 Hz was applied, in accordance for standard procedures described by Koenig et al [25]. Next, microstate clusters were then produced using the *Compute Microstates* function. Here we selected to produce only 4-clusters (maximum = 8) with a convergence criterion set to 25 iterations. All maps produced here were polarity insensitive and were only generated at GFP peaks. After this process was repeated for all 237 cases, the resultant average microstate maps (N = 237) were entered into the *Combine Microstates* function which clustered all of the scalp maps of each individual to produce 4 mean microstate-classes; the classes were almost identical to those reported by Koenig et al. [25] and explained approximately 72% of the variance in scalp map classification.

Next, 4 microstate statistics (occurrence, coverage and duration) for each microstate class as well as model percent variance (which effectively described how well the average model fit the individual subjects) were computed within the software. To accomplish this all 4-second segments (of each participant) were re-imported into Mapwin software. The average 4-class model computed above was then applied to each of these segments (N = 237x4) separately and statistics were computed using the *Microstate Statistics* function which exported each statistic for each epoch into a Microsoft Excel file. Averages of each of the statistics for each individual were then computed.

Finally, the raw voltages, spectral densities, inferences of parahippocamapal activity, and microstate parameters were exported into a singular SPSS dataset for further analyses.

## Study 2-Real Time Correlations Between Schumann resonance Measured Locally and Non-Locally and Brain Spectral Power Densities

To replicate our previous findings [11] and those of Pobachenko et al [12] indicating a synchrony between signals measured from the earth-ionospheric cavity and electroencephalographic activity two participants (2 male and 2 female) were recruited to participate. While the relationship has since been replicated with a larger population involving synchronous correlations between brain activity and the Schumann resonance measured in Italy, the effect sizes for the relationship between Schumann and human brain coherences made locally were such that we decided two randomly selected individuals should be demonstrative; in effect the correlations observed within these two individuals should be comparable to that observed in a previous study [11].

### Local Brain-Schumann Coherence

Two individuals (1 male and 1 female) were outfitted with a 19-channel QEEG cap (Electro-Cap International) which was connected to a Mitsar-201 amplifier while laying in a supine position outside the Laurentian University Arboretum, which was distant enough from sources of electrical interference (i.e. power and telephone lines) to allow for relatively noise-free recordings. All sensor impedances were maintained below 5 kOhms and were sampled at 500 Hz within WinEEG 2.8 software. A lead from a custom-constructed induction coil magnetometer system was fed into the ECG input of the amplifier in order to allow simultaneous recordings of brain electrical activity and electromagnetic perturbations occurring within the local earth-ionosphere.

The exact specifications and methodology of the experiment and equipment (including pictures) were identical to a protocol previously described [11]. For brevity, the coil was composed of 28 gauge copper wire (approximately 14 kg) wound around a 5-cm diameter PVC pipe and was connected into a pre-amplifier with a gain of 40 dB. Calibration with the Mitsar box demonstrated that the magnetometer was sensitive to changes on the order of a picoTesla and on clear and cloudy days with minimal wind Schumann resonances were readily observable in the averaged spectrum after 1–2 minutes of continuous sampling. This is similar to the time required by other researchers [26] to clearly discriminate the Schumann power densities. In addition the variations in power within the Schumann signals from our station are strongly correlated with those of the on-line data from an Italian station (Observatory IK1QFK; www.vlf.it).

For both participants, the caudal root-mean-square was calculated from the 7 posterior channels (T5,P3,Pz,P4,T6,O1,O2) as already described for 60-second segments (N = 2) where the Schumann resonance was readily observable from initial spectral screening. This signal as well as the magnetic component of extremely-low frequency (ELF) activity collected simultaneously were then merged together into a two-channel set and entered into EEGLab [19] for channel cross-coherence for estimations of coherence magnitude and phase. This measure was computed in 10-second time bins using sinusoidal Hanning-tapered wavelets 3 cycles in length 0.5 seconds long and a padding ratio of 1.

### Non-Local Brain-Schumann Coherence

To discern whether the coherence observed between the Schumann resonance and brain electrical activity was similar when ‘atmospheric noise’ was recorded non-locally, a stream of ultra-low frequency electric field perturbations (http://78.46.38.217/vlf15.m3u), measured with a Marconi T antenna, was directed into the Mistar ECG input using a custom-constructed audio-to-ECG cable for 2 subjects (1 male and 1 female). The stream is made available from Mr. Renato Romero who owns and operates an open-source website dedicated to the exchange of techniques and real-time radio frequency data below 22 kHz for amateur radio hobbyists. One-way ping times were measured from this site from our laboratory in Sudbury and the results indicated that the maximum latency was 50 milliseconds. Data collection and analysis was exactly the same as for the two participants whose ELF and EEG data were measured locally; the only difference was that five minutes of eyes closed data was collected for 30 participants within a sound-proof acoustic chamber instead of outside.

## Results

### Differential Potential Differences

**Fig 1:**
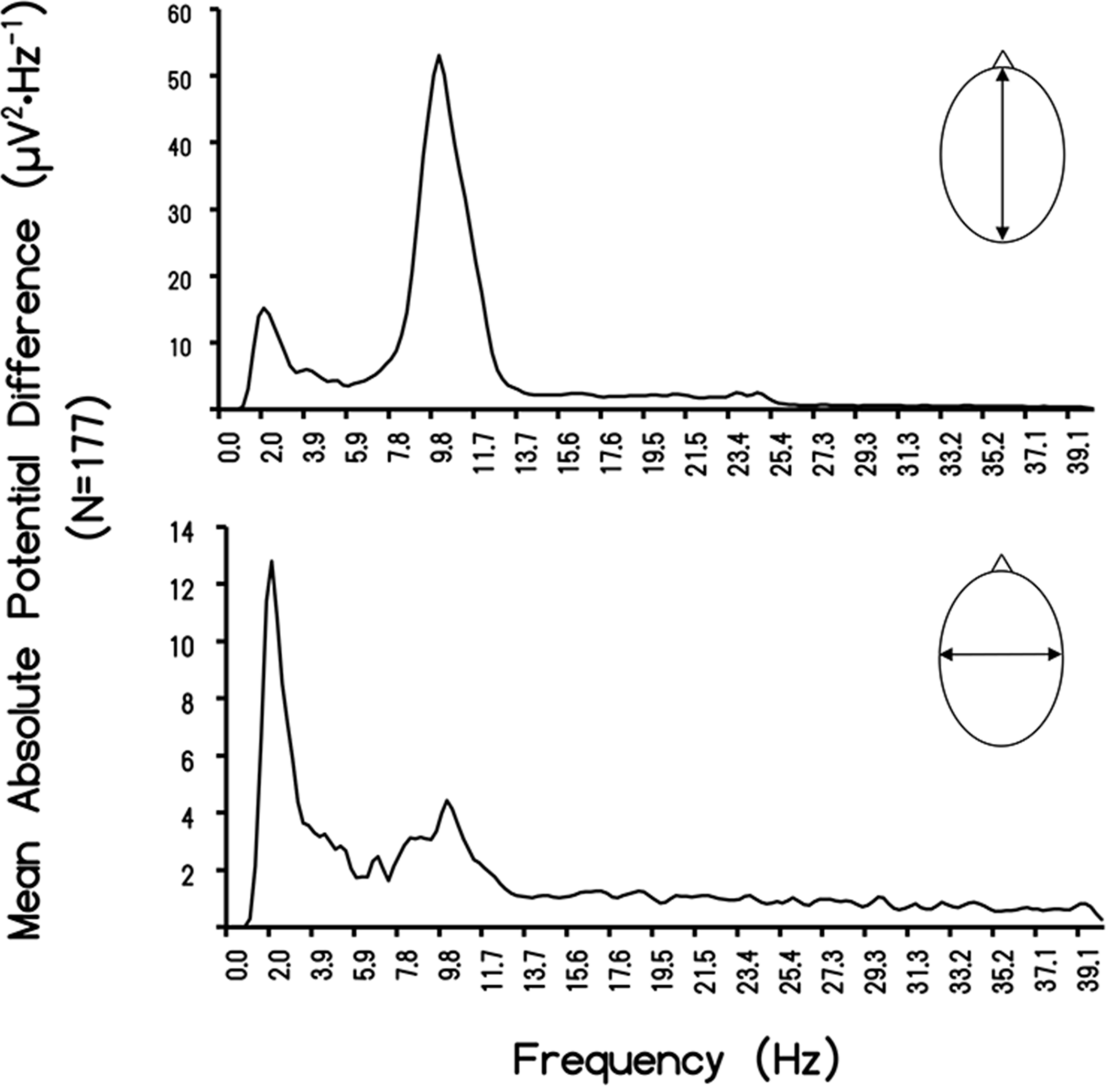
Rostral-Caudal Difference. Absolute potential difference (ΔμV^2·^Hz^-1^) plotted as a function of frequency for the rostral-caudal and left-right measurements during QEEG for 177 subjects measured over a three year period.

**Fig 2:**
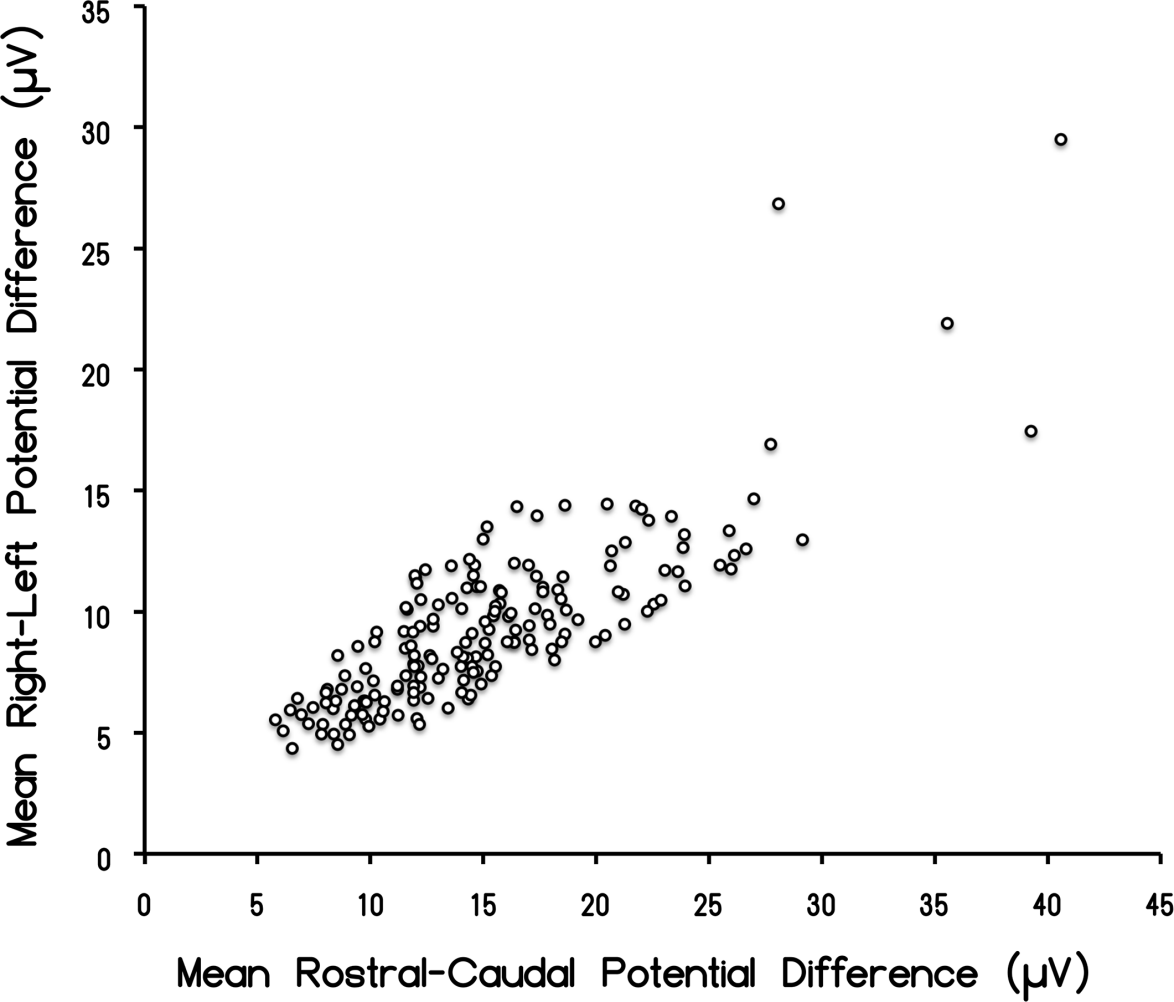
Rostral-Caudal Correlation. Scattergram of the correlation (r = 0.82) between the discrete potential difference values for the caudal-rostral vs left-right potential differences.

The means for the μV^2^/Hz for global frequencies between the rostral-caudal (RC) axis and the left-right (LR) temporal lobe for the 237 records available for this study are shown in Fig 1. For this particular analysis (N = 177), we only selected individuals whose z-score was within +/- one standard deviation from the mean. The amount of shared variance (r^2^) was 67% (i.e., r = 0.82). After accommodating for bandwidths pertaining to the window size of the spectral analysis, the square root of the two mean values was 3.89 μV and 3.05 μV, respectively, or a major (RC)-to-minor (LR) axis ratio of 1.27. According to Blenkov and Glazer [27] the mean rostral-caudal length of the cerebrum is 172.5 mm (155–190 mm) while the RL length is 136 mm (131–141). The ratio is 1.27. Consequently the average disparity of potential difference between the two axes reflects the proportional distance between the sensors. The scattergram displaying the correlation between the discrete quantitative values for the RC and LR measurements is shown in Fig 2.

### Results of Random Sample (n = 10) Analyses

**Fig 3:**
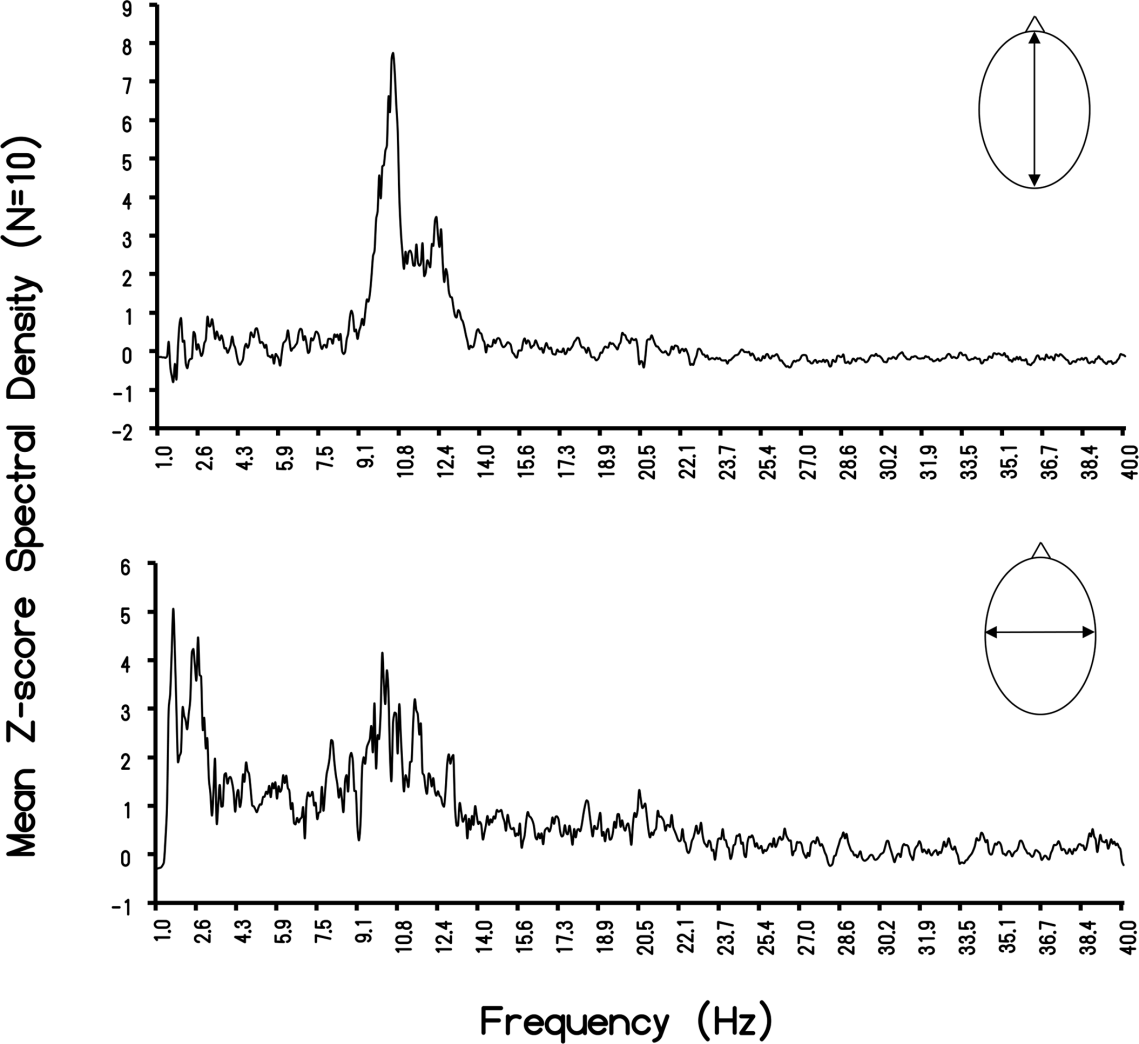
Rostral-Caudal and Left-Right Individual Differences. Means of the z-scores of the spectral density (μV^2^·Hz^-1^) of the measurements as a function of frequency for the rostral-caudal and left-right measurements.

The correlation of the voltage values between the RC and LR axes for the 10 subjects was r = 0.88. Because the values were μV^2^·Hz^-1^, square root values were obtained for the FC value (3.71±0.76 μV) and RL (2.88±0.47 μV). The ratio was 1.29, compared to the ratio of 1.27 for the entire sample. The distributions of the spectral density profiles for the major and minor axes of the human cerebra in our sample are shown in Fig 3. The classic peak around 10 Hz, with the majority of the power within the area of the curve between ~7 Hz and 13 Hz, is evident. The mean for this population was 10.25 Hz. Although the later peak was evident for the minor (LR) axis, most of the power density for the LR axis was around 2 Hz (1.95 Hz) with a more leptokurtic boundary within the delta band. The primary beat (difference) frequency was 8.3 Hz.

More detailed examination of the power spectra for the 10 randomly sampled records employed to verify RC-LR differences in voltage revealed potentially relevant individual variability. Employing the peak z-range obtained from each of the increments of frequency from the spectral analyses, the mean peak was 10.12 Hz for the RC and 2.44 Hz for the LR axis. The primary beat (difference) was 7.7 Hz. The results of analyzing the “width” of the peak in spectral power densities are shown in Table 1 for each of the 10 subjects. The mean frequency of the “peak band width” was 0.39 Hz.

**Table 1:** Peak Frequency Individual Differences. Range of the standardized (z-score) for the raw spectral density values for the peak band (in Hz) for the 10 randomly selected subjects. The “band width” for the peak band range is also shown.

Subject	Z-Range	Peak band (Hz)	Bandwidth (Hz)
1	12–14	11.43–11.68	0.25
2	10–14	10.18–10.75	0.57
3	8–14	10.31–10.50	0.19
4	10–11	12.18–12.43	0.25
5	3–6	10.51–11.31	0.8
6	9–19	10.31–10.68	0.37
7	15–18	10.43–10.68	0.25
8	2–4	11.00–11.37, 11.75–12.50	0.37, 0.75
9	6–19	9.75–10.18	0.43
10	9–15	11.93–12.31	0.38

### The Schumann Resonance Signature

**Fig 4:**
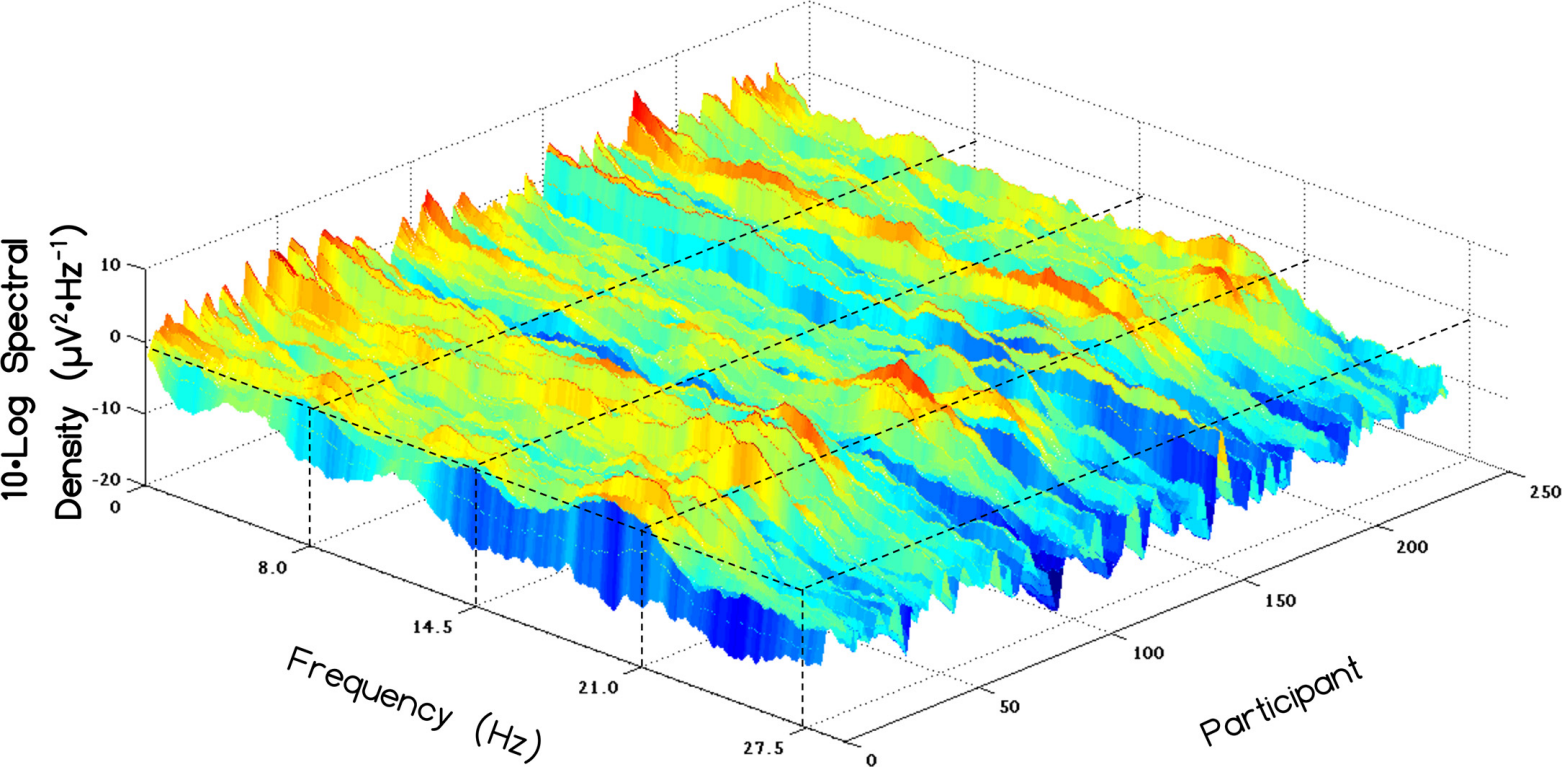
Individual Electroencephalographic Schumann Resonance Profiles. Log of spectral density of various frequencies reflecting the Schumann resonance for all 237 records.

A spectral distribution comparable to the Schumann resonances recorded from the earth was observed with peak frequencies centering at approximately 7.5 Hz, 13.5 Hz and 20 Hz within the global QEEG power for 237 records. One outlier (with a z>10) was removed. A 3-D plot showing the distribution of each individual's spectral profile for the caudal aspect is displayed in Fig 4.

We reasoned that if this resonance pattern within the human brain was normally distributed, certain individuals should show elevations in the spectral density of the Schumann resonance. Thus, we computed a measure of Schumann intensity by multiplying the spectral density of the mean of 7–8 Hz, 13–14 Hz and 19–20 Hz spectral densities for each of the rostral, middle, and caudal RMS values described above. This computation provided an overall measure of the strength of the Schumann resonance signature found within the cerebrums of all 237 records along the rostral, middle, and caudal regions of the brain. These values were then cube-rooted for each signal (i.e. rostral, middle and caudal) separately. Hence the equation characterizing the computations for each of the 3 signals could be described by:

$$Mean(x)={\left[x(7-8\;Hz\;Density)\times x(13-14\;Hz\;Density)\times x(19-20\;Hz\;Density)\right]}^{1/3}$$

Where x corresponds to the selected region of computation (i.e. rostral, middle or caudal).

We then computed a global measure of the Schumann resonance by averaging all 3 (rostral, middle and caudal) of these newly computed measures. Individuals were then categorized into one of the 3 groups based upon the z-scores of this global average. The groups were low (z<-.5), medium (-.5<z < .5) and high (z>.5). This classification resulted in 19 individuals (M = 11, F = 8) within the low group and 34 individuals (M = 9, F = 25) within the high group; the rest of the individuals were within the medium group (M = 89, F = 95).

**Fig 5:**
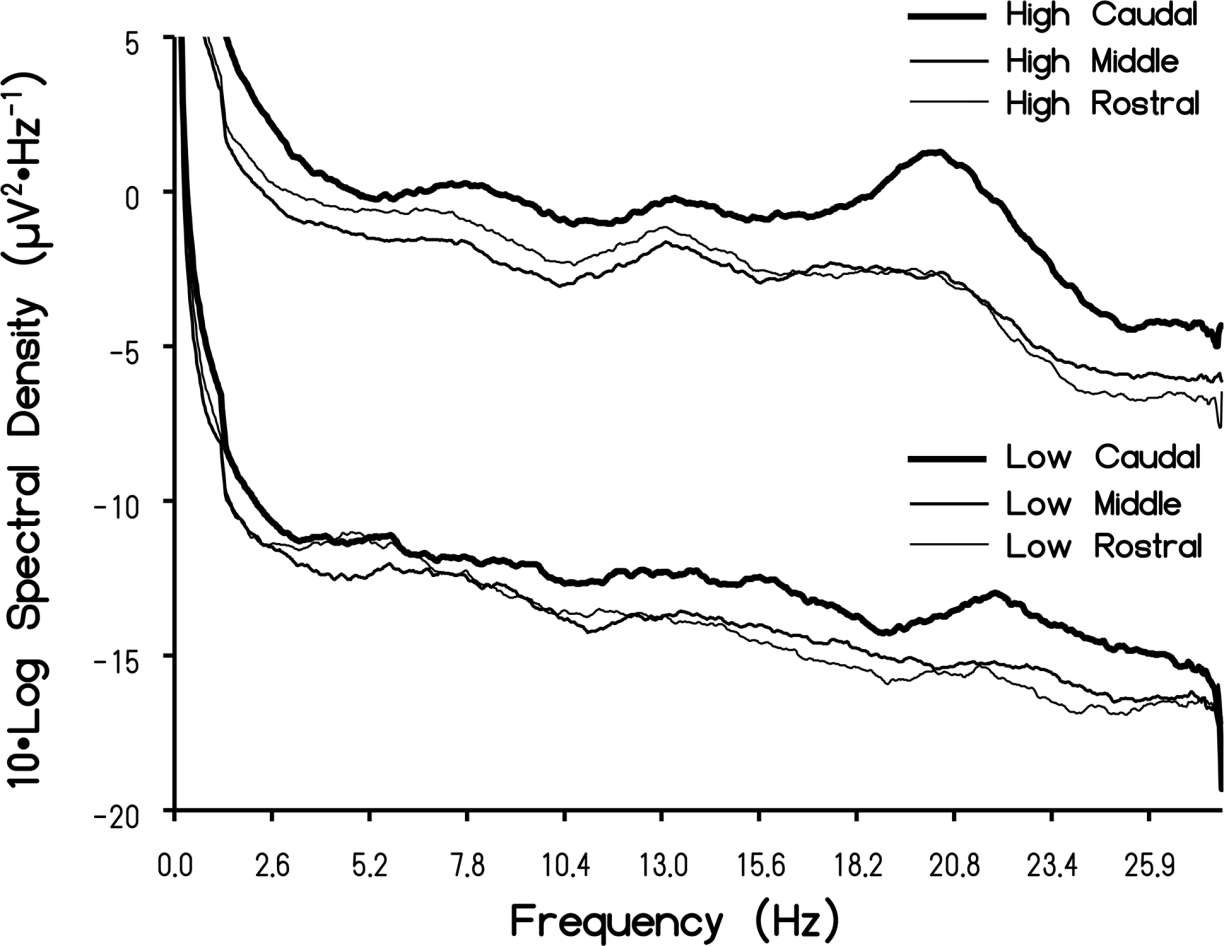
Averaged Schumann Resonance Profiles. Mean Log value of the spectral density for high and low intensity subjects as a function of frequency for the rostral, middle, and caudal regions of the cerebrums. Note the peaks at the fundamental Schumann resonance (first harmonic) as well as the second (about 14 Hz) and third (about 20 Hz) harmonics.

When the discrete spectral scores for each group were averaged across individuals within a given intensity group (high versus low) and graphed, there were qualitative differences in the definition of the spectral profiles (which were smoothed with a moving average with a period = 40 FFT points, or approximately 1 Hz). Fig 5 depicts the spectral profiles within the rostral, middle and caudal regions as a function of intensity group (low Schumann intensity versus high Schumann intensity). The first observation was that the Schumann resonance was more defined for individuals who displayed stronger Schumann resonances intensities, defined by their z-score. Whereas the low group could be described as "flat", the high group showed peaks and valleys more characteristic of standing waves of 7.18 Hz, 13.52 Hz and 20.29 Hz. The transformed difference for the high and low group was ~1 μV and 0.3 μV per Hz, respectively. The peak to trough difference at the 20 Hz peak for the high spectral density group would be about 2 μV^2^ ∙ Hz^-1^. The second observation was that, regardless of group (high versus low intensity), the Schumann resonance was more pronounced within the caudal regions of the brain.

### Selected Associations Between the Schumann Resonance Signature with Other Measures of Brain Activity

Given the remarkable rhythmic character of the subthreshold oscillation of ~8 Hz for stellate cells in Layer II of the entorhinal cortices [28] and the importance of this region for receiving convergent inputs from the entire cortical manifold before their distribution to the hippocampal formation and amygdala, we specifically investigated the parahippocampal region by sLORETA using the three computed Schumann intensity groups. Separate one-way analyses of variance for the left and right parahippocampal current source densities within the pre-defined frequency bands with Schumann intensity (low, medium, high) as a between-subject factor indicated that there was significantly (p < .001) more current source density within the bilateral parahippocampus across most frequency bands for individuals who displayed high Schumann resonance intensities. The strongest effects, as inferred by effect size, were observed within the theta and gamma frequency ranges within the left hemisphere. The relationship appeared linear which suggested that as the endogenous Schumann Resonance intensity increased the activity in the left parahippocampus increased. These results are shown in Table 2.

**Table 2:** Means and Standard Errors of the Means (in parentheses) for current source density (μA·mm^-2^) of the parahippocampal gyrus within the classical frequency bands.

	Left				Right			
	Low	Medium	High	Effect Size	Low	Medium	High	Effect Size
Delta	737 (67)	1412 (81)	1999 (257)	0.06	678 (64)	1563 (112)	1856 (230)	0.04
Theta	122 (11)	341 (22)	866 (147)	0.19	115 (9)	408 (35)	897 (150)	0.12
Low Alpha	188 (38)	1005 (90)	2710 (642)	0.12	149 (26)	1277(116)	2888 (495)	0.13
High Alpha	238 (54)	847 (71)	1435 (158)	0.09	279 (77)	951 (82)	1988 (272)	0.12
Beta-1	352 (50)	619 (40)	1134 (117)	0.12	345 (50)	667 (44)	1206 (142)	0.11
Beta-2	189 (49)	248 (26)	452 (62)	0.05	166 (32)	285 (37)	429 (45)	0.02
Beta-3	589 (224)	512 (93)	1188(421)	0.03	471 (174)	499 (74)	918 (206)	0.02
Gamma	2649 (319)	5783 (280)	11356 (1088)	0.23	2419 (239)	6608 (350)	12004 (893)	0.2

**Fig 6:**
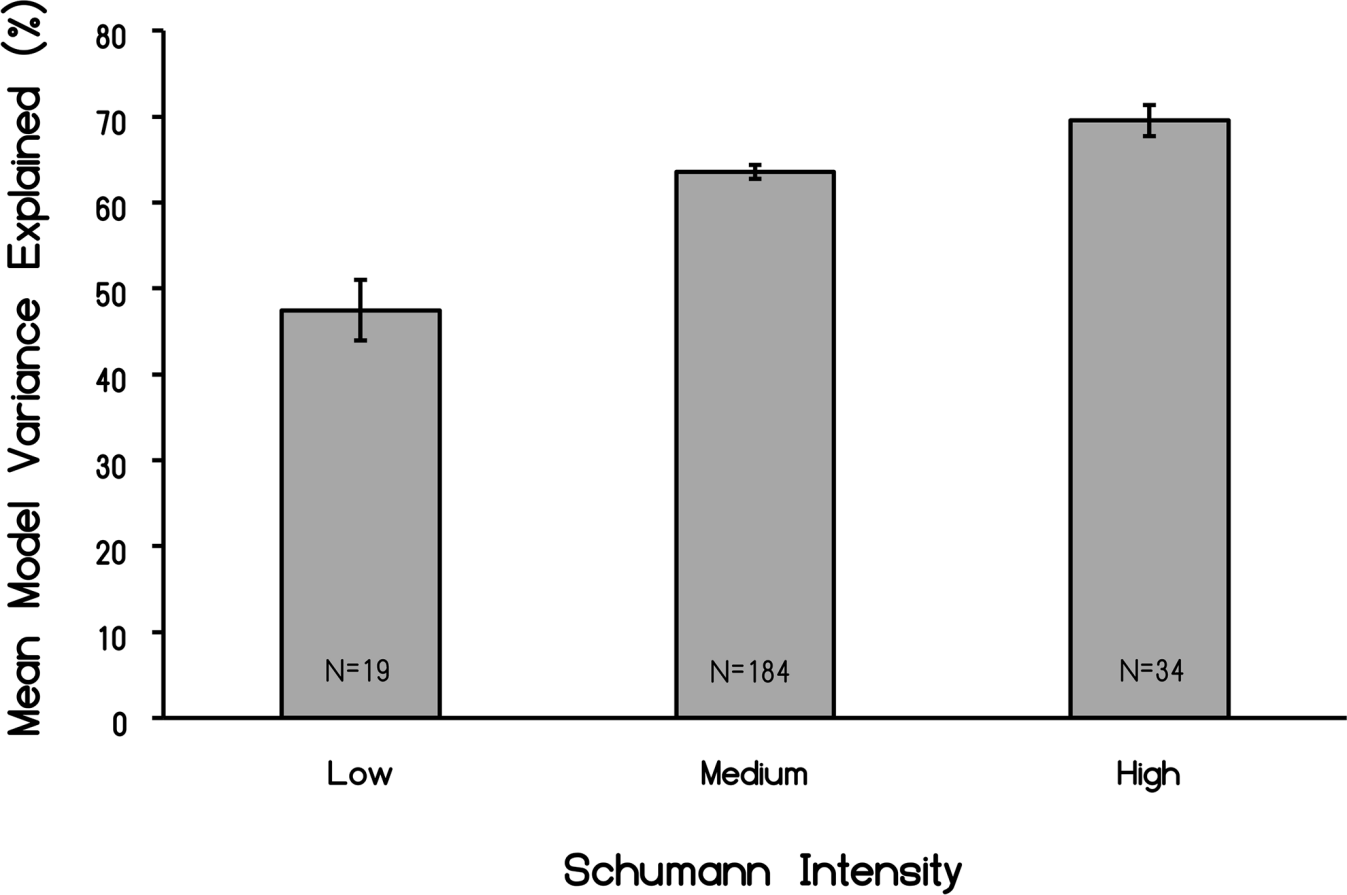
Variance explained of all classic microstates as a function of the intensity of the Schumann resonance (first three harmonics) within the EEG. N indicates numbers of records per *post hoc* group.

We also decided to explore this categorization of Schumann intensities within the context of microstate topographies, which may be considered as geometric shapes of the electric field (and by association magnetic field) produced by constructive and destructive interference processes. A one-way analysis of variance indicated that the model percent variance of the four microstate clusters explained significantly more variance (F_2,234_ = 22.89, p < .001, Ω^2^ estimate = .16) in brain topographical patterns within individuals who displayed “strong” Schumann resonances. These results are shown in Fig 6 and indicated that the classic 4-class model, used to describe normal resting-state EEG topographies, was highly influenced by the presence of the Schumann resonance within human brain cortical activity.

### Real Time Coherence Between Directly Measured Schumann Resonance Power Densities and QEEG Profiles

**Fig 7:**
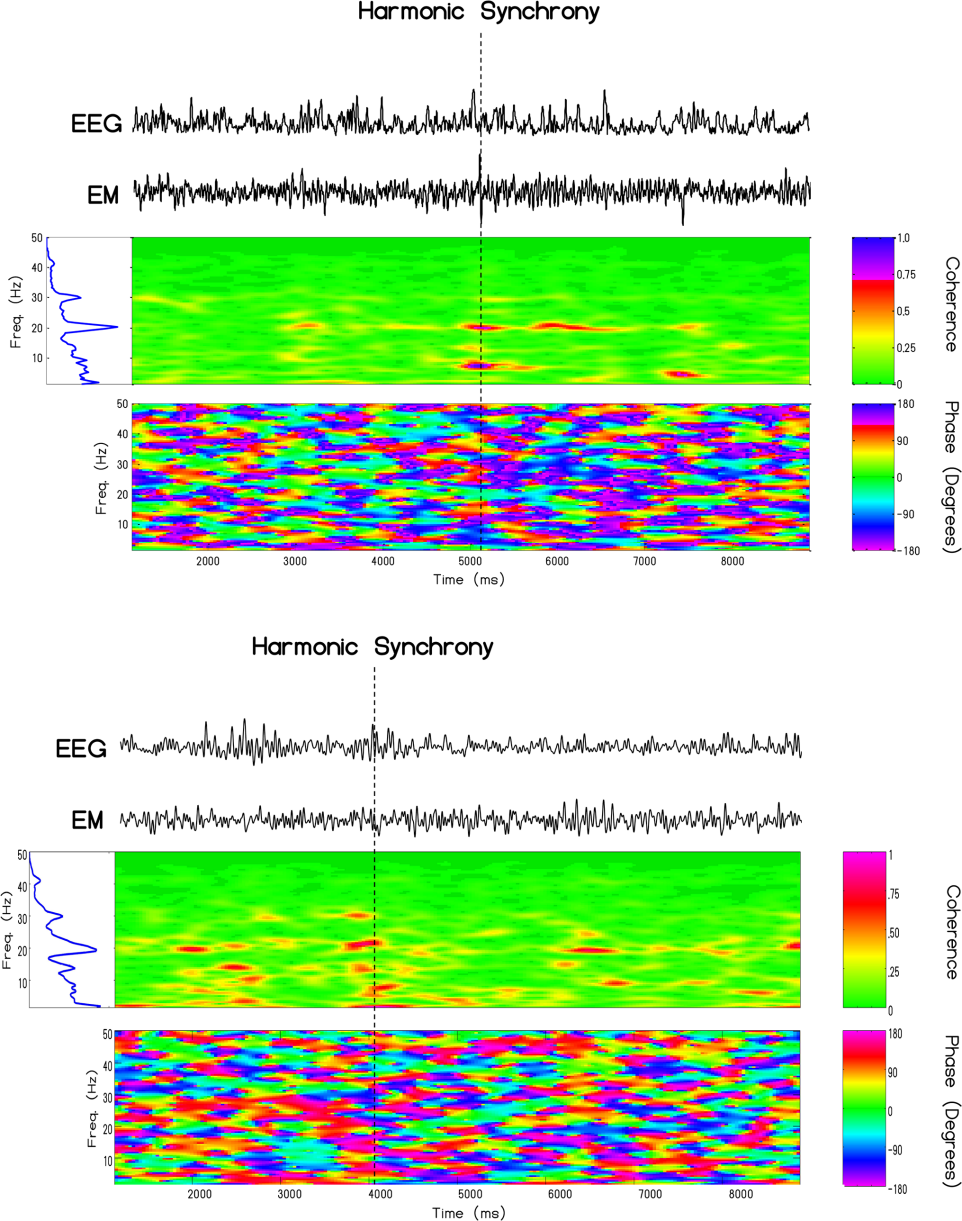
Local Harmonic Synchrony. Cross-channel coherence between the caudal root-mean-square derivation and extremely low-frequency electromagnetic activity recorded simultaneously in Sudbury, Canada for one male and one female measured on separate days. Evident in this time-frequency analysis is harmonic synchrony occurring between brain electrical activity and atmospheric ‘noise’ at approximately 8, 13 and 20 Hz which define the first three harmonics of the Schumann resonance.

The coherence profiles for two subjects (1 male and 1 female) whose QEEG were measured and spectral analyzed at the *same time* as direct measurements were taken for the Schumann Resonance locally and within 1 meter of the person are shown in Fig 7. For the first person the major coherence, as indicated by the red color that reflects the highest correlation occurred for intervals of about 300 ms for the frequencies 8 Hz, 13 Hz, and 20 Hz. The greatest phase shift was at 7.8 Hz and was equivalent to about 38.1 ms. For the second person the congruence between the person’s spectral power density for brain activity and the simultaneous Schumann measurements were strongest within the same three frequency bands. The phase shift at 7.8 Hz for that person on that day was 57 ms. The results for these two people are very similar to the pattern we observed for a third person [11]. They are also consistent with the data reported by Pobachenko et al [12] whose measurements were completed in Russia and with the general properties of phase-modulation [26].

**Fig 8:**
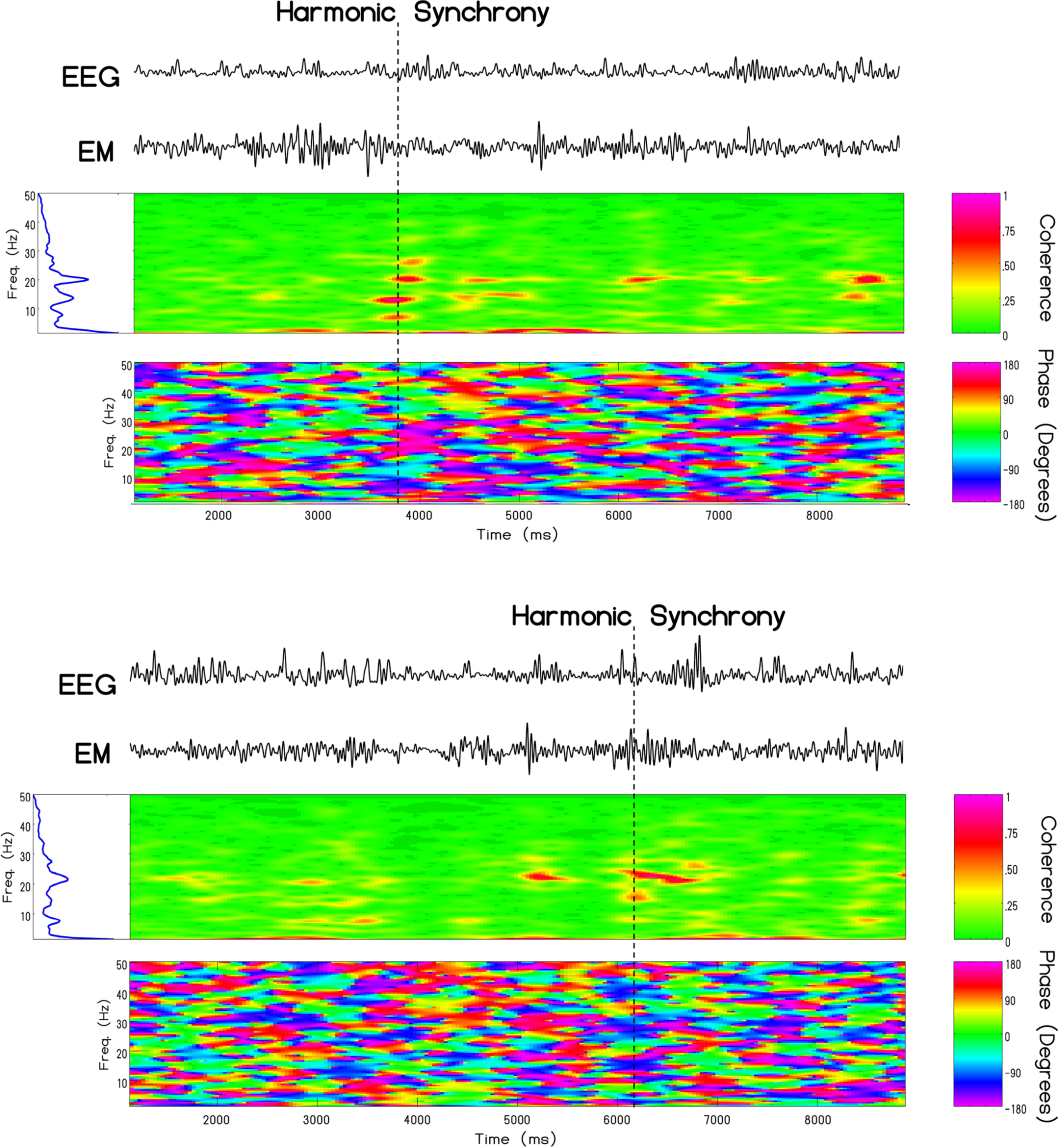
Non-local Harmonic Synchrony. Similar harmonic synchrony between extremely low-frequency atmospheric noise measured in Cumiana, Italy and brain electrical activity measured in Sudbury, Canada at 8, 13, and 20 Hz for two subjects (1 male and 1 female) measured on a different day.

Similarly, the results of the same cross-channel coherence analysis indicated periods of harmonic synchrony between the cRMS and ELF recorded in Italy. Maximal synchronization was commonly observed simultaneously within the 7–8 Hz, 13–14Hz and 19–20Hz frequency bands, although scattering of coherence amongst other frequencies was common. Fig 8 displays exemplary cases of harmonic synchrony for a different male and female. As in the cases where the Schumann resonance was monitored locally, harmonic synchronous events were approximately 300 milliseconds in duration and typically occurred about 1–2 times per minute.

## Discussion

Our results confirm and extend the results from other researchers that the QEEG properties of the human brain reflect the subtle differences in volume and three-dimensionality [5], the peaks of spectral power of the earth’s Schumann resonances in many (but not all) individuals [3] and the real time coherence between the spectral power densities [12]. For the brain parameters, the absence of statistically significant differences between the strengths of the correlations of power between the rostral-caudal and left-right comparisons and their ratios of power for the total sample and the random sub-sample of 10 subjects indicates the robust nature of these relationships. The precision of the ratio of the physical differences in RC and LR distances of a population of adult cerebrums and the differences in the power densities of electrical activity support our disciplines assumption that the amplitude of QEEG activity is a physical reflection of potential difference (voltage).

### Quantitative Inference of Cerebral Magnetic Field Strength

We reasoned that if the cerebral and earth-ionospheric resonances shared some characteristic that could allow potential interaction, some variant of “diffusivity” should be involved. Magnetic diffusivity, ή = (μ_o_σ)^-1^–, where μ_o_ is magnetic susceptibility (4π∙10^−7^ N∙A^-2^) and σ = electrical conductivity, is a concept relevant to geophysical [29] and neurophysical phenomena. Assuming the average resistivity of 2 Ω∙m (σ~ 0.5 S·m^-1^) for extracellular fluid, and the average potential difference of 3 μV in the rostral-caudal axis observed here, the resulting estimate for the magnetic field strength is (3∙10^−6^ kg∙m^2^∙A^-1^·s^-3^) divided by 1.58∙10^6^ m^2^∙s^-1^ or ~1.9∙10^−12^ T, that is about 2 picoTeslas (pT). This is well within the range based upon direct measurement from of action potentials from isolated frog sciatic nerves at distance of 1 mm [30]. The spatial domain is sufficiently large to have major influence upon the integrated cerebral field. This magnitude is within a factor of 10 of the transient magnetic oscillatory response [31] evoked in the human cerebrum, with a peak near 10 Hz and 30–40 Hz, when the measured values in 20 fT∙Hz^-1/2^ are applied over the full frequency range of 100 Hz.

Although these intensities appear small, experimental application of pT, spatial and temporally-patterned magnetic fields have been reported to produce discernable improvement in some clinical disorders, such as Tourette’s and multiple sclerosis [32,33] as well as some electrical foci associated with partial seizures [34]. Correlative responses associated with subjects’ experiences have been associated with geomagnetic changes in the order of pT·s^-1^ [35]. The latter occurred when ambient geomagnetic changes displayed progressive increases at this rate over an approximately 15 min interval.

### Comparisons with the Fundamental Schumann Resonance and Interhemispheric Beats

It may be relevant that the recondite “beat” (the difference between the frequencies) between the rostral-caudal and left-right power peaks was between 7 Hz and 8 Hz, the range of the fundamental (first harmonic) of the Schumann resonance. This compliments the more global QEEG emergence of the first, second and third harmonics when a different method of analysis was employed. The intrinsic “resonance frequency” mediated through the “external shell” of the cerebral cortices [14] is also within this interval. Precise frequencies in this range can be important. For example Harmony [36] found that 7.8 Hz power within the frontal lobes decreased in adults but increased in children during a task that emphasized short-term memory.

Resonances often arise in a cavity with sharp, homogeneous, and isotropic boundaries. The Schumann resonances, with a fundamental frequency around 7.8 Hz and harmonics around 14.1, 20.3, 26.4, and 32.5 Hz [16], are generated within the approximately 100 km earth-ionospheric cavity. The higher modes (harmonics) are separated by ~6 Hz according to the general formula *f*_n_ = *f*_1_[(n(n+1)]^1/2^ although some authors include geometric coefficients. Primary origins of the energy are attributed to global lightning activity [7]. The typical median value for the electric field is ~1 mV m^-1^ while the magnetic field strengths are in the order of 1 pT. At the lowest mode, around 8 Hz, the attenuation is ~1 db per 2000 km [16]. The significance of these “weak amplitudes” become salient to neuroscience when one realizes that the potential differences involved with reconnection of flux lines within the geomagnetic field when solar wind energy is transferred into the magnetosphere is actually about 4 μV·cm^-1^ [37] which is within cerebral parameters.

Although popular authors emphasize the exactness of the fundamental (first harmonic) frequency, empirical measurements indicate a range that would be significant from a QEEG perspective. For example injections of energies from protons (solar proton events) into the upper atmosphere are followed by systematic increases of between 0.04 to 0.14 Hz above the fundamental (7.5 Hz) and amplitude enhancements of between 0.11 and 0.42 pT relative to the 1 pT background [17]. In addition, splitting of Schumann resonances have been measured intermittently for decades [38]. While pursuing the theoretical predictions of Madden et al [39], Tanahashi [40] noted doublet and triplet “split” peaks at 7.1, 7.85 and 8.5 Hz for the first mode and 12.4, 13.3, 14.0 and 14.8 Hz in the second mode. Fraser-Smith and Buxton [38] showed that doublets (8 and 10 Hz) occurred for several tens of minutes. The amplitudes of these discrete peaks were in the order of 1 or 2 pT·Hz^-1/2^.

Analyses of global power of 16-second samples of QEEG data from almost 200 subjects over a five-year period indicated that spectral densities exhibited relatively wide-band peaks within the first three harmonics of the Schumann resonance. The origins of these fundamental and harmonic enhancements with the QEEG data cannot be identified at this time. One possibility is that the Schumann peaks may reflect some intrinsic feature of the cerebral space due to the average circumference and bulk velocity [6,14] or even a coupling between cerebral and ionospheric frequencies through mechanisms yet to be identified. If the latter condition is valid then intrinsic interactions, most likely intermittent, might occur and could be potentially quantified by near-future QEEG and source-location technology. Transience may define these phenomena within QEEG profiles. In our studies the harmonics were not obvious within single 16-s samples but were clear when aggregates from many subjects were averaged. Interestingly this process is also required to obtain spectral profiles (such as in Fig 5 for brain activity) even with direct environmental measurement of Schumann resonances [26]. It is also noteworthy that optimal filtering between 1.5–40 Hz was required in order to observe Schumann frequency peaks. When the low-cut frequency was set to below 1.5 Hz or above 3 Hz, the characteristic peaks were not observed. This may indicate that energies represented within the 1.5–3Hz frequency band are crucial in discerning the Schumann resonance within the brain.

### Individual Differences

Although the statement that individual differences is the largest source of variance is well known to any first year psychology student, the relevance to electrophysiological measurements was obscured until QEEG became the general tool. If there is intercalation between exogenous sources of Schumann resonances and comparable global cerebral frequencies then quantifying and classifying these features may help explain sensitive populations. The individual differences were remarkable. In Table 1 the z-score for the peak band in the spectral densities for each person are shown. A representation of the 7.5 Hz Schumann line in brain activity would occur in only two of the 10 subjects. Line splitting of the Schumann resonance displays peaks with frequency spacing between ~ 0.2 to ~0.6 Hz. This overlaps with the bandwidth of the peak power frequency. That only about 10% to 13% of all brains showed enhancements in the intensity of the Schumann resonance may be useful for defining the characteristics of this subpopulation.

There were also individual differences (or more accurately group differences) for the amount of variance in the four classic microstates as a function of the intrinsic power of the first three harmonics of the Schumann frequencies within the cerebral volume. As the power for these bands increased the percentage of variance explaining the four microstates increased from about 48% to 68%. Or stated alternatively, less of the total variance for the classic microstates was explained for those brains that displayed the lowest Schumann spectral density. This could be interpreted as Schumann power functions like a “homogenizer” by removing “random” or “unique” sources of variation and enhancing the prevalence of the four basic microstates. Considering its global nature, a direct contribution of Schumann resonance-induced power to the proportional predominance of the four major microstates if they are the building blocks of thought [41], could potentially affect large numbers of the human species for transient periods.

### Potential Interactions Between Human Brain and Schumann Dynamics

Whether or not information from the cerebral volume could be represented within the Schumann volume (or visa versa) has significant philosophical and social implications that QEEG and neuroimaging could soon address. Even relatively complex mathematical solutions suggest that gravity waves would interface with the earth’s second harmonic (14 Hz) Schumann resonance [42]. The energy associated with the loss or gain of a bit of information according to the Landauer Limit (ln 2 kT where k is the Boltzmann constant and T is temperature) is equivalent to about 2.97∙10^−21^ J, at brain temperature. In comparison, the energy upon a unit charge from the ~2.6 (±0.5) mV of stellate cells that occurs within the upper (Layer II) cortical neurons within the entorhinal cortices or parahippocampal region [28], which is 4.16∙10^−22^ J would be precisely within this range because this energy is generated about 8 times per second (~8 Hz) by the persistent subthreshold oscillations. The stellate cells in the medial entorhinal cortices are involved with convergences of information from the entire cortices and are a primary origin of the perforant path fibers to the dentate gyrus of the hippocampal formation.

Our results indicated that the average current source density within the parahippocampal regions was greatest within the *theta* and *gamma* bands for individuals who displayed elevated amplitudes for the three first harmonics of the Schumann resonance. This may be relevant not only because the “gamma” or “40 Hz” ripples are superimposed upon hippocampal theta oscillations [43]. The interconnectedness between hippocampal theta and cerebral cortical gamma activity has been considered an important electrophysiological correlate for the intercalation between neurocognitive processes such as consciousness and memory [44]. Theta phase can modify the synchrony or coherence between gamma oscillations originating in multiple regions. It may also be relevant that the hippocampal formation and the parahippocampal regions, particularly within the right hemisphere appear, to be particularly responsive to small changes in geomagnetic activity and experimentally applied fields within the pT range [9,32,33,35].

Clearly the most compelling evidence that the similarities between the intensities of the magnetic field and electric field strengths generated within the earth-ionosphere cavity, measured locally in Canada and non-locally in Italy, and those measured by EEG from the human brain as well as the appearance of Schumann resonances within the EEG profiles of a large population of subjects that we have measured was the real-time coherence. Pobachenko et al [12] had measured such coherence, employing different methods, in a different area of the world. The enhanced coherence of power spectral between the simultaneous Schumann powers within the first three harmonics and these frequencies within the EEG of the subjects is consistent with a direct relation between the two. The coherence is not continuous but occurs in quasi-discrete durations in the range of 300–400 ms and approximate that of the single microstate. Even the phase-modulations of between 40 to 60 ms reflects the band width ratios. For example (Table 1) the ~0.4 Hz width divided by the primary beat frequency (7.7 Hz) would be equivalent to a proportion that is about 50 ms.

Although Koenig et al [2,3] may have argued that the Schumann patterns are the zeitgebers for this real-time correlation, there may be third factors that affect both. Pobachenko et al [12] noted that both the Schumann and EEG profiles varied with changes in geomagnetic activity. Saroka et al [10] measured greater EEG coherence between left and right temporal lobe structures during increased geomagnetic activity. The effect sizes for the coherences exhibited maxima for the fundamental and first harmonic of the Schumann Resonance. That geomagnetic activity affects QEEG profiles has been shown by correlation [9] and experimental [45] analyses.

One of the basic assumptions of modern Neuroscience is that ultimately all of the qualitative features of the ephemeral cognitive processes such as thinking and consciousness will be interpretable as *quantitative configurations* [46] of the complex temporal patterns of electromagnetic values that can be measured from the surface of the human cerebrum. The similarity of average duration of microstates and the duration of the human percept is an example of such transformation of perspective. The clever consideration [47,41] that four microstates [25] could reflect functional cognitive units or “atoms of thought” that *are* the information within the stream of dynamic process within cerebral space, analogous to base nucleotide pairs for genetic information within DNA sequences, exemplifies the significance of this approach. The shift towards biophysical analyses [6,14,48,49,50] of complex cerebral functions once relegated to the domain of the philosopher and psychologist requires a verification of the physical parameters for these operations. Here we reiterate and report validation of quantitative phenomena that define the electric and potentially magnetic features of our primary inferential measurement: quantitative electroencephalography (QEEG) and how their characteristics are shared by a unique pervasive property of the earth-ionosphere cavity.
